# Activation Likelihood Estimation Meta-Analysis of the Effects of Cognitive Behavioral Therapy on Brain Activation in the Treatment of Depression and Anxiety Disorders

**DOI:** 10.1155/da/3557367

**Published:** 2025-06-17

**Authors:** Junjie Ren, Lijun Ma, Wanxin Wu, Juan Qiu, Zhuo Zhang, Yuxi Hong, Zushen Wang, Xinyu Hou, Jia Jin, Weixuan Hu, Yanran Wu, Xiaowei Chen, Jinyi Luo, Chuan Fan, Kai Wang, Xiaoming Li

**Affiliations:** ^1^Department of Psychiatry, Chaohu Hospital of Anhui Medical University, Hefei, Anhui, China; ^2^Department of Medical Psychology, School of Mental Health and Psychological Science, Anhui Medical University, Hefei, China; ^3^Department of Maternal and Child and Adolescent Health, School of Public Health, MOE Key Laboratory of Population Health Across Life Cycle, Anhui Provincial Key Laboratory of Population Health and Aristogenics, Anhui Medical University, Hefei, Anhui, China; ^4^Department of Psychiatry, the First Affiliated Hospital of Anhui Medical University, Hefei 230022, China; ^5^Anhui Province Key Laboratory of Cognition and Neuropsychiatric Disorders, Hefei, China; ^6^Collaborative Innovation Center of Neuropsychiatric Disorders and Mental Health, Hefei, China; ^7^Institute of Artificial Intelligence, Hefei Comprehensive National Science Center, Hefei, China

**Keywords:** anxiety disorders, cognitive behavioral therapy, depressive disorders, meta-analysis

## Abstract

**Background:** Cognitive behavioral therapy (CBT) stands as a highly efficacious psychological treatment for both anxiety and depressive disorders. Nonetheless, scholarly debates persist regarding the specificities of brain area activation during CBT treatment for these disorders.

**Methodology:** Utilizing activation likelihood estimation (ALE) analysis, this study aims to discern the neurobiological similarities and disparities between CBT's effects on anxiety and depressive disorders by examining functional brain areas.

**Results:** A total of 22 articles, encompassing 443 patients, were included in the meta-analysis. Our results show that in the resting state, patients with depression treated with CBT resulted in increased activation of the right and left ventral anterior cingulate cortex (vACC), left parahippocampal gyrus (PG), right subgyral, left inferior temporal gyrus (ITG), and right inferior occipital gyrus (IOG), whereas patients with anxiety disorders had increased activation of the right and left superior frontal gyrus (SFG) and decreased activation of the caudate after treatment. In the task state, increased activation of the right PG, right orbital frontal lobe, and right dorsal anterior cingulate cortex (dACC) was mainly observed after treatment in patients with anxiety disorders, and the left lentiform nucleus (LN), left dorsal entorhinal cortex, and right caudate activation were decreased. For depressive disorders, no consistent activation patterns emerged in the task state, likely due to limited studies or heterogeneity in task paradigms across included studies.

**Conclusion:** CBT's efficacy relies on both shared (e.g., vACC-mediated emotion regulation and cognitive control) and distinct neural mechanisms (fear-circuit modulation in anxiety vs. memory-network enhancement in depression), informing biomarker-driven treatment personalization.

## 1. Introduction

Anxiety and depressive disorders are two pervasive mental health conditions with distinct but often overlapping symptoms. According to the American Psychological Association, anxiety manifests as emotional discomfort and physical tension, typically involving activation of the autonomic nervous system [1]. Numerous studies have shown that anxiety disorders are associated with the onset and progression of cardiac disease and, in many cases, with adverse cardiovascular outcomes, including mortality [2, 3]. Depression, another prevalent condition, is characterized by enduring feelings of sadness, anhedonia, and diminished motivation.

A comprehensive review by Santomauro et al. [4] assessed the impact of the COVID-19 pandemic on the rates of depression and anxiety disorders across 204 countries and territories from January 2020 to January 2021. Excluding the influence of the pandemic, the global prevalence rates of depression and anxiety in 2020 would have been approximately 2470.5 and 3824.9 per 100,000 individuals, respectively [4, 5]. According to an assessment by the World Health Organization, approximately 280 million people in the world suffer from depression, while 301 million people globally suffer from anxiety disorders [6]. In addition, depression is associated with an elevated risk of suicide, with more than 700,000 deaths by suicide each year, making it a mental health issue of high clinical concern [7]. Identifying effective treatments for mental health conditions like anxiety and depression is of paramount importance. Cognitive behavioral therapy (CBT) serves as a frontline intervention for these disorders, rooted in cognitive and behavioral psychological theories. This therapy equips patients with the tools to understand and reframe their thought patterns and behaviors, thereby mitigating or resolving their emotional challenges [8]. CBT for these disorders is organized into distinct phases, including cognitive restructuring, behavioral experimentation, and problem-solving [9].

During therapy sessions, the clinician collaboratively engages with the patient to explore their cognitive landscape and behavioral tendencies. The aim is to identify detrimental thought patterns and dysfunctional behaviors [10]. Subsequently, the therapist guides the patient through cognitive and behavioral adjustments, providing targeted psychological support and facilitating effective change.

CBT's applicability is vast; it is a widely acknowledged and empirically supported treatment for depression [11]. In contrast to mindfulness-based cognitive therapy (MBCT), which focuses on accepting negative thoughts and emotions rather than directly challenging them [12], CBT focuses on identifying and modifying negative cognitions. Additionally, CBT has shown promise as a short-term, highly effective intervention compared to psychodynamic therapy and acceptance and commitment therapy for a range of anxiety disorders, including obsessive–compulsive disorder (OCD), generalized anxiety disorder, and panic disorder [13].

The advent of advanced brain imaging techniques has provided a biological foundation for the emotional, cognitive, and behavioral transformations facilitated by psychotherapy. For example, studies using positron emission tomography (PET) have identified increased activation in the right posterior cingulate gyrus (PCC) in patients with panic disorder following CBT treatment [14]. These investigations not only offer biomarkers that validate the efficacy of psychotherapy but also contribute to the broader understanding and acceptance of psychotherapeutic interventions for various psychological disorders. Nonetheless, debates persist about the mechanisms underlying psychotherapies, including CBT. Despite overarching similarities, most psychotherapies exhibit unique theoretical and practical attributes that warrant further exploration [15].

Numerous studies have demonstrated that CBT induces discernible changes in both structural and functional brain images for patients with depressive and anxiety disorders. For instance, Kennedy et al. [16] observed decreased metabolic levels in the PCC among patients with depressive disorders post-CBT, a finding echoed by Burklund et al. [17] in patients with social phobia. Nevertheless, the literature exhibits two key inconsistencies: first, while some studies identify activation in the same brain regions following CBT, the directionality of these changes varies. For example, in the resting task, Goldapple et al. [18] found elevated blood oxygen levels in the parahippocampal gyrus (PG) in post-treatment depressive patients, whereas Sankar et al. [19] reported a decrease. Second, certain studies have reported alterations in brain regions that others have not identified. For example, in the fear experience task, Paquette et al. [20] found changes in the dorsolateral prefrontal cortex (dLPFC) and the PG in spider phobia patients following CBT treatment, whereas Schienle et al. [21] detected changes in the medial orbitofrontal cortex in patients with spider phobia post-CBT.

Addressing these discrepancies and identifying both commonalities and differences in brain imaging outcomes between CBT treatments for depressive and anxiety disorders remain critical questions. Meta-analyses may offer a means to consolidate findings across multiple studies, thereby providing more robust conclusions.

Sankar et al. [22] carried out a meta-analysis investigating the neural underpinnings of psychotherapeutic interventions, including CBT, for major depression. The study revealed reduced activation in the left precentral gyrus among patients post-therapy. Similarly, Picó-Pérez et al. [23] conducted a meta-analysis focusing on the neural correlates of CBT in treating anxiety-related disorders, identifying that changes in fronto-insular (right inferior frontal gyrus, anterior insular cortex) and fronto-limbic (dorsomedial prefrontal cortex, dorsal anterior cingulate cortex [dACC]) cortices were strongly correlated with successful CBT outcomes. While these meta-analyses lend credence to the efficacy of CBT through pooled effect sizes and co-activation changes in specific brain regions, several challenges remain. First, the extant literature on the neural correlates of CBT for depressive or anxiety disorders is sparse. Many of the studies in this domain suffer from limited neuroimaging data, a small sample size, and heterogeneous criteria for subject inclusion. Second, discrepancies in diagnostic criteria across different meta-analyses add an additional layer of complexity. For instance, Picó-Pérez et al. [23] utilized the DSM-IV to categorize OCD and post-traumatic stress disorder (PTSD) as anxiety disorders, a classification not universally adopted in other meta-analyses. Lastly, a significant gap in the literature is the absence of studies that explore the neural correlates of CBT for both depressive and anxiety disorders in a comparative framework. This omission is particularly noteworthy given the symptomatic overlap and high co-morbidity between these disorders [24], as well as the effectiveness of similar treatments for both conditions [25].

In the current meta-analysis, we conducted a pioneering investigation into the neural correlates of CBT in the treatment of depressive and anxiety disorders. For the first time, this study encompassed functional imaging, including resting and task states. Our sample was confined to cases of depressive and anxiety disorders and included only studies employing neuroimaging techniques. The findings from this meta-analysis aim to deepen our understanding of the mechanisms of action underlying CBT's efficacy in treating these prevalent mental health conditions. This research contributes to elucidating the neural substrates involved and may thereby facilitate more targeted and effective interventions for both depressive and anxiety disorders.

## 2. Methods

### 2.1. Literature Search and Inclusion

A comprehensive literature search was executed across multiple databases, including PubMed, ClinicalTrials, Embase, Web of Science, Cochrane, Ovid Medline, and PsycINFO, to identify studies published between January 2000 and January 1, 2023, that investigated changes in brain activation among patients with depressive and anxiety disorders undergoing CBT. The search was refined using a set of predetermined terms, such as “major depressive disorder,” “psychotic depression,” “dysthymia,” “anxiety disorder,” “MRI,” “specific phobias,” “social phobia,” “panic disorder,” “social anxiety disorder,” “fMRI,” “neuroimaging,” and “cognitive behavior therapy” (see Table S1 for the complete search strategy). To supplement this search, pertinent reviews [26] and meta-analyses [22, 23, 27, 28] were manually scrutinized to ensure that no relevant publications were overlooked. Additionally, correspondence was initiated with study authors for clarification and to inquire about the availability of supporting data. This meta-analysis was conducted in rigorous accordance with the PRISMA guidelines, and its protocol has been registered with PROSPERO, the international prospective register of systematic reviews and meta-analyses (CRD42022377058).

Inclusion criteria: (1) Study population: The research must focus on patients diagnosed with either depressive disorder or anxiety disorder, without limitations on age, gender, or source of recruitment; (2) treatment modality: The study must employ CBT, which includes variations, such as group CBT (GCBT) and internet CBT (ICBT), with a treatment duration of no less than 4 weeks; (3) sample size: The study should have a treatment group comprising more than five participants; (4) neuroimaging techniques: Studies must utilize neuroimaging methods, such as functional magnetic resonance imaging (fMRI), PET, or diffusion tensor imaging (DTI). Additionally, the studies should assess and report activation in specific brain regions; (5) reporting standards: Studies are required to delineate changes in brain regions before and after treatment. These alterations should be specified in a stereotactic space, utilizing three-dimensional coordinates (*x*, *y*, *z*) to pinpoint the locations of activity; and (6) assessment timing: Neuroimaging assessments must be performed both at baseline, prior to the initiation of treatment, and subsequent to the treatment period.

Exclusion criteria: (1) Co-morbidity and drug use: Studies with patients having comorbidities or recent medication use before CBT will be excluded. All participants should be free of psychotropic medications. No participants tested positive for drugs; (2) case studies: Individual case studies will not be considered for inclusion; (3) impure CBT therapies: Studies employing therapies that are variants of CBT, such as MBCT or standalone cognitive therapy, were excluded. These approaches differ from standard CBT in theoretical focus and techniques (e.g., MBCT emphasizes mindfulness rather than behavioral activation), potentially leading to distinct neural effects [12, 29]; (4) review and meta-analysis: Literature reviews and meta-analyses are not eligible for inclusion; (5) incomplete reporting: Studies that do not provide coordinates for areas where neuroimaging changes occurred will be excluded; and (6) concurrent treatments: Studies involving patients who are undergoing other forms of therapy or treatment concurrently will not be considered.

### 2.2. Data Extraction

Key variables, such as demographic characteristics, treatment durations, neuroimaging tasks, and primary study outcomes, were meticulously extracted. Additionally, neuroimaging data were further detailed, encompassing sample size, normalization templates (either MNI or Talairach), spatial coordinates (*x*, *y*, and *z*) for specific brain regions, and the field of view, which could range from a designated region of interest (ROI) to the entire brain. The first data was extracted on May 3, 2023. Literature searches, inclusion criteria evaluations, and data extraction procedures were independently conducted by Junjie Ren and Wanxin Wu and subsequently reviewed for accuracy and consistency by the corresponding author.

### 2.3. Meta-Analysis

The activation likelihood estimation (ALE) meta-analysis technique was initially formulated by Turkeltaub et al. [30]. This method calculates the probability of voxel activation under specific experimental conditions and compiles these probabilities. We employed GingerALE 3.0.2, a specialized software for conducting ALE meta-analysis within BrainMap, to analyze coordinates in either Talairach or MNI space [31]. Three-dimensional brain coordinates indicating treatment-induced changes were extracted from comparisons made within the treatment groups before and after therapy. Given that our meta-analysis was performed in the standard MNI coordinate space, results initially reported in Talairach space were converted to MNI coordinates by using a nonlinear transformation approach [32]. In alignment with recommendations from the ALE guidebook, we established the significance threshold for the ALE at *p*  < 0.001. This threshold was further corrected by employing the method of uncorrected *p*-values [32], with a minimum cluster size defined as 200 mm^3^ [33].

To elucidate the similarities and differences in brain activation changes following CBT treatment for patients with depressive and anxiety disorders, we conducted several meta-analyses segregated by functional imaging categories. Specifically, we performed meta-analyses to evaluate: (1) alterations in brain regions in the resting state of patients with both disorders post-CBT; (2) changes occurring in the task state for these patients post-treatment; and (3) consistent shifts in brain regions across resting state and task state after treatment for both depressive and anxiety disorders. In addition, we analyzed subgroups according to the same task paradigm and type of anxiety disorder. Each meta-analysis generated ALE result maps and summary cluster reports. These reports detailed the clusters of brain region activations, along with their peak ALE values, signifying the probability of activation in those respective regions [34]. To enhance the visual representation of our findings, we employed Mango software (http://ric.uthscsa.edu/mango/) to overlay the extracted coordinates onto a standardized brain template [35]. This facilitated a graphical depiction of the brain regions' activation clusters.

### 2.4. Analyses of Quality Assessment

So far, there has been no standard checklist for assessing the quality of individual functional neuroimaging studies. A checklist, which included assessment of participants, image acquisition and analysis methods, and results and conclusions, with 10 subcategories, from a previous meta-analysis was utilized [36]. The total score ranged from 0 to 10. The risk of bias was classified as high (1–3), medium (4–7), and low (8–10). The quality assessment was conducted based on standardized items (Table S2). If there was disagreement between two primary reviewers regarding the study's quality, a supervisor independently assessed the article.

## 3. Results

### 3.1. Characteristics of Included Studies

**Figure 1:**
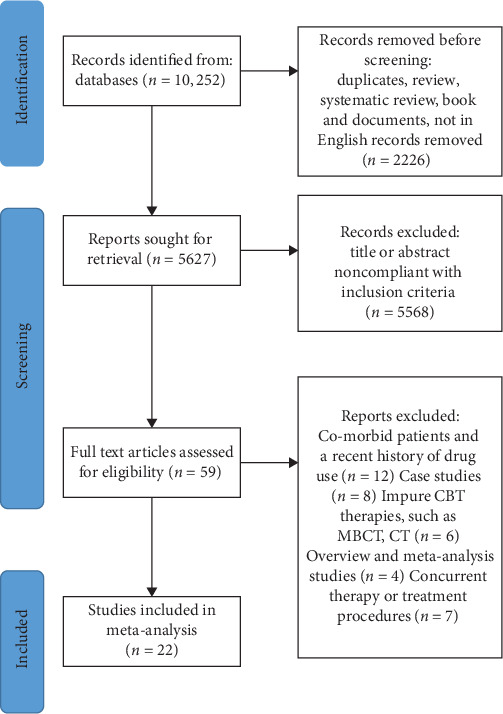
Preferred Reporting Items for Systematic Reviews and Meta-Analyses (PRISMA) flow diagram of search strategy.

The literature search identified 10,252 studies and 2226 duplicates; after a review of abstracts and titles, 59 studies were included for detailed assessment, of which 22 met our inclusion criteria. The search process is shown in Figure 1.

The main meta-analysis included 22 studies that met the inclusion and exclusion criteria, consisting of a total of 135 patients with depressive disorders and 308 patients with anxiety disorders. All studies used DSM or ICD criteria to diagnose depressive disorders and anxiety disorders, and all had significant treatment effects from CBT (Table S3). Of the 22 included studies, six of them contained two or more treatment groups, were treated with different methods, and reported pre- and post-treatment changes in each group of subjects separately [17, 18, 37–40].

Categorizing by imaging methodology, 16 studies employed fMRI, and six utilized PET. Of the functional studies, six examined resting-state imaging and 16 investigated task-state imaging. Additionally, when segregated by disorder type, nine studies targeted depressive disorders, and 13 explored anxiety disorders.  Table 1 displays the specific traits of the included studies.

### 3.2. Main Meta-Analysis

### 3.3. Results of Brain Area Activation in the Resting State

In this meta-analysis, a total of 6 studies employing resting-state imaging were analyzed. For depressive disorders, nine sites exhibited increased activity, and 19 sites showed decreased activity. For anxiety disorders, there were 21 sites of increased activity and 12 sites of decreased activity. The ALE meta-analysis results revealed that in the resting state, six clusters of increased activation, specifically in the right ventral anterior cingulate cortex (vACC), the left vACC (BA24), the left PG (BA4), the right subgyral, the left inferior temporal gyrus (ITG), and the right inferior occipital gyrus (IOG), were identified in patients with depressive disorders who underwent CBT treatment (Table S4 and Figure S1). No clusters of decreased activity were observed, likely due to the limited number of included studies. Conversely, for patients with anxiety disorders receiving CBT, one cluster of decreased activation in the caudate and two clusters of increased activation in the left superior frontal gyrus (SFG) and the right SFG (BA10) were found (Table S5 and Figure S2). These results indicate that CBT induces distinct neural activation patterns in the resting state for depression and anxiety. In depression, increased vACC and PG activation suggest CBT improves symptoms by enhancing cognitive control and reshaping emotional memory [54]. Conversely, in anxiety, increased SFG activation and decreased caudate activity suggest CBT acts by bolstering prefrontal modulation of fear and reducing automatic threat responses [55].

**Figure 2:**
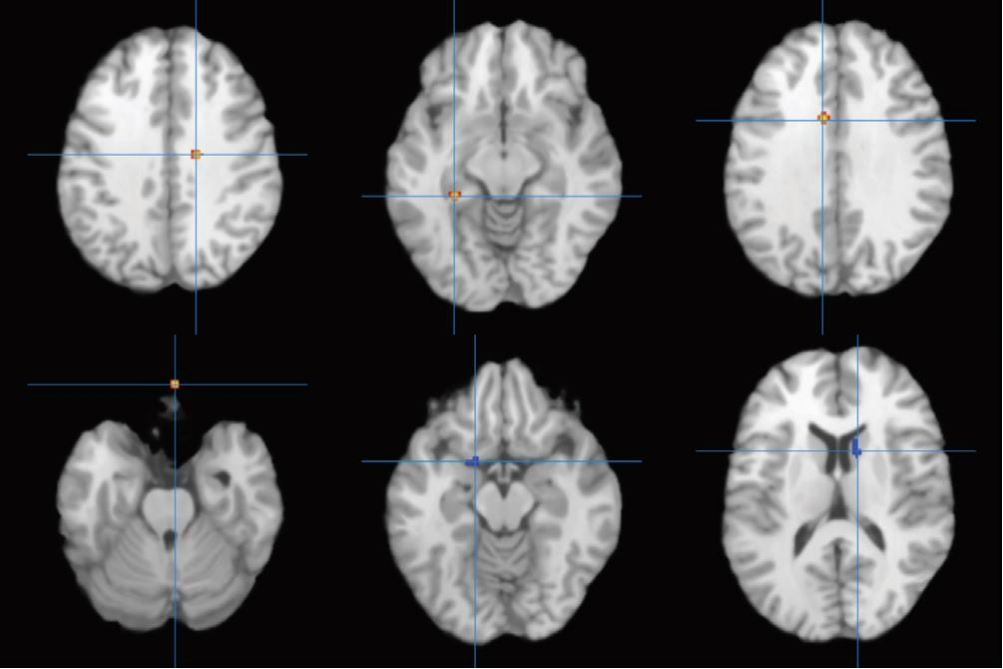
Graph of data meta-analysis results for different imaging states. Uncorrected *p<*0.001, min. cluster size = 200 mm^3^, the brain regions activated in the figure reached significant activation levels. The red activation area is the activity increase area and the blue activation area is the activity decrease area. The brain regions activated in the diagram are, from left to right: row 1: the right ventral anterior cingulate cortex (BA24), perirhinal cortex, the left ventral anterior cingulate cortex (BA24) and row 2: orbital frontal lobe (BA11), lentiform nucleus, caudate.

In addition, the ALE meta-analysis results revealed that in the resting state, three clusters of co-increased activation, specifically in the right vACC (BA24), the perirhinal cortex (BA36), and the left vACC (BA24), were identified in patients with depressive and anxiety disorders who underwent CBT treatment. Comprehensive details are provided in Table S6 and illustrated in Figure 2.

### 3.4. Results of Brain Area Activation in the Task State

In this meta-analysis, a total of 16 studies using task-state imaging were analyzed. For depressive disorders, 23 sites exhibited increased activity, and 37 sites showed decreased activity. For anxiety disorders, there were 14 sites of increased activity and 62 sites of decreased activity. For patients with anxiety disorders receiving CBT, three clusters of increased activation in the right orbital frontal lobe, the right PG, and the right dACC and three clusters of decreased activation in the left lentiform nucleus (LN), the right caudate, and the left dorsal entorhinal cortex (BA34) were found (Table S7 and Figure S3). However, for patients with depressive disorders treated with CBT in the task state, we did not observe any alterations in brain region activation. This could be attributed to the limited number of studies included, as well as the varied task paradigms utilized in the included studies.

In addition, the ALE meta-analysis results revealed that in the task state, two clusters of co-decreased activation, specifically in the left LN and the right caudate and one co-increased activation, specifically in the right OFC, were identified in patients with depressive and anxiety disorders who underwent CBT treatment. Comprehensive details are provided in Table S6 and illustrated in Figure 2.

For patients with anxiety disorders treated with CBT in the task state, we analyzed subgroups by type of anxiety disorder as well as by task paradigm. For social anxiety disorder, one cluster of decreased activation in the LN was found. For specific phobia, five clusters of increased activation in the right OFC, the right superior parietal lobule (SPL), the left SPL (BA7), the right IFG (BA9), and the right dACC (BA32) were found. Comprehensive details are provided in Table S8. For the emotional face task, two clusters of increased activation in the right dACC (BA32) and the left paracentral lobule (BA4) were found (Table S9). For the fear experience task, all subjects had specific phobias. Five clusters of increased activation in the right middle occipital gyrus (BA18), the right SPL (BA7), the left SPL (BA7), the right IFG (BA9), and the right dACC (BA32) and three clusters of decreased activation in the left thalamus, the right thalamus, and the left dorsal entorhinal cortex (BA34) were found (Table S10).

### 3.5. Quality Assessment

The scores obtained were all above 9, indicating the high quality of the papers included in the meta-analysis. The quality assessment scores can be found in the supporting information (refer to Table S11).

## 4. Discussion

This study systematically elucidates the differential and shared neural mechanisms of CBT in treating anxiety and depressive disorders through meta-analysis. By integrating 22 neuroimaging studies (*n* = 443), we found that CBT consistently enhances cognitive control function in the vACC and reduces limbic system (e.g., caudate) hyperactivation in both disorders, supporting its transdiagnostic efficacy. Importantly, disorder-specific effects were observed: for anxiety disorders, CBT primarily modulated fear circuits (increased dACC/OFC activation and decreased LN response during task states), whereas for depressive disorders, it significantly enhanced memory-related functions in the PG. Furthermore, neural changes in depression were more prominent during the resting state, while anxiety disorders showed more pronounced task-state effects, suggesting distinct temporal dynamics in treatment response. These findings not only provide neurobiological explanations for CBT's clinical effectiveness but also offer critical evidence for developing personalized treatment protocols based on neural functional characteristics.

### 4.1. Different Changes in Brain Area Activation in CBT for Depressive Disorders and Anxiety Disorders in the Resting State

The findings of this study reveal that CBT exerts different effects on the activation patterns of specific brain regions in patients with depressive and anxiety disorders in the resting state. For patients with depression, we observed increased activation in key regions, such as the right and left vACC, the PG, the ITG, the IOG, and the subgyral. In contrast, for patients with anxiety disorders, we observed increased activation of the left and right SFG as well as decreased activation of the Caudate. These outcomes offer novel insights into the neurobiological mechanisms that underpin the efficacy of CBT.

For patients with depression, firstly, our findings indicate a significant modulation of activity in the PG following CBT treatment. This observation is consistent with existing neuroimaging literature, which highlights the PG's critical role in emotional memory [54]. Aberrant activity in this region has been linked to deficits in emotion regulation [56]. Thus, it can be posited that CBT may enhance emotional memory function in patients with depressive disorders by modulating activity in the PG.

Secondly, our study revealed that CBT notably influenced activity levels in the ITG and IOG. The ITG, part of the default mode network (DMN), is involved in cognitive processes and contributes to processing emotional stimuli [57]. Prior research has shown a marked decrease in network homogeneity and brain region activation of the ITG in depressed individuals [58]. While for the IOG, it is primarily involved in visual processing and facial expression recognition, and serves as a pivotal region in the brain's visual recognition circuitry [59].

Furthermore, CBT influenced activity in the subgyral. Previous studies have found that the subgyral activation is significantly lower in the resting state than in healthy controls [60]. As a part of the temporal lobe, the sub-gyral is primarily associated with cognitive and emotional regulation [61]. CBT is a well-established psychotherapeutic intervention that is effective in correcting maladaptive thinking patterns and enhancing emotional self-regulation.

For patients with anxiety disorders, our findings indicate a significant modulation of activity in the left and right SFG following CBT treatment. The SFG is situated in the medial frontal lobe and forms part of the prefrontal lobe [55]. It plays a crucial role in the cerebral cortex and is linked to various cognitive functions and emotional regulation [62]. The SFG is implicated in higher cognitive processes like decision-making, planning, executive control, and emotion regulation. Moreover, it is connected to social cognition and emotional processing, including emotion recognition, regulation, and social interaction [55]. A study revealed that SFG activation in individuals with GAD was connected to the therapeutic benefits of CBT [63]. However, given the diversity of anxiety disorders, further investigation is necessary to fully understand the potential of CBT for the SFG.

In summary, our meta-analytic findings suggest a nuanced relationship between the therapeutic mechanisms of CBT in treating anxiety and depressive disorders and the associated changes in brain activation patterns in the resting state. To better understand these observations and to elucidate their clinical implications, further research is warranted.

### 4.2. Different Changes in Brain Area Activation in CBT for Depressive Disorders and Anxiety Disorders in the Task State

The findings of this study reveal that CBT exerts different effects on the activation patterns of specific brain regions in patients with depressive and anxiety disorders in the task state. For patients with anxiety disorders, we observed increased activation in key regions, such as the right OFC, the PG, the dACC and decreased activation in the LN, the caudate, and the dorsal entorhinal cortex. However, for patients with depressive disorders, due to the limited number of included studies and the fact that the included studies utilized different task paradigms, we did not observe any changes in brain region activation.

Our findings indicate a significant modulation of activity in the LN following CBT treatment. The LN is implicated in a range of functions, including emotion regulation, cognitive control, and stress response [64]. Existing literature posits that the LN is functionally compromised in individuals with anxiety disorders, marked by a reduced neuronal density [65]. Further, a meta-analytic review has shown that activity in the LN is elevated in states of anxiety [62]. The diminished activation of the LN observed in our study could suggest that CBT effectively mitigates its hyperactivity, thereby potentially ameliorating symptoms of anxiety and depression in patients. This modulation of LN activity may serve as a pivotal neurobiological mechanism through which CBT exerts its therapeutic effects on anxiety disorders.

Furthermore, CBT influenced activity in multiple brain regions, including the right dACC, the OFC, and the dorsal entorhinal cortices. These areas are pivotal in memory control, decision-making, and emotion regulation, functions that are often impaired in patients with anxiety disorders [66]. For instance, the dorsal entorhinal cortices play an important role in spatial memory and navigation, especially in the construction of cognitive maps and spatial localization [67], while the OFC is essential for emotion regulation and decision-making processes [68]. Consequently, it is plausible that CBT may alleviate symptoms of anxiety by normalizing functions within these critical regions.

**Figure 3:**
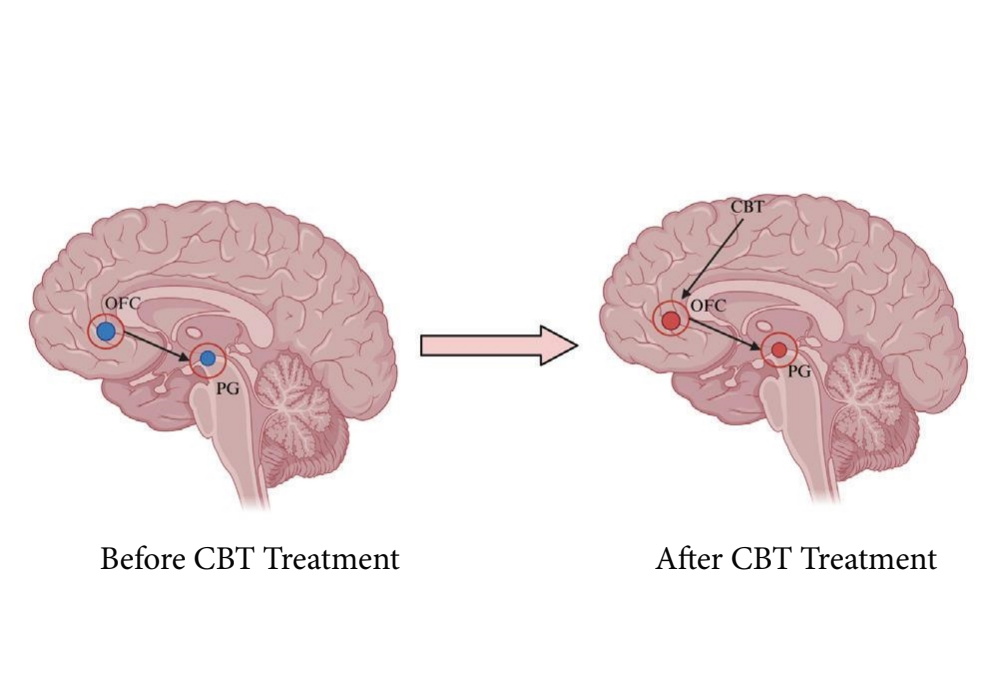
Graph of the mechanism of action of CBT efficacy based on emotional neural circuitry. Before treatment, patients with anxiety disorders and depressive disorders had abnormally low activation in the orbital frontal lobe (BA11), parahippocampal gyrus, and CBT treatment increased parahippocampal gyrus activity (top–down) by increasing orbital frontal lobe (BA11) activity. The red activation area is the activity increase area and the blue activation area is the activity decrease area. PG, parahippocampal gyrus.

Our findings indicate that CBT is crucial for enhancing emotional circuits. Recent research on the emotional brain has shown that emotions are regulated by a neural circuit in the brain and that the different brain regions in the circuit are interconnected rather than operating independently [69]. For example, increased activity in the ACC was found to suppress activity in the caudate nucleus [70]. In light of our meta-analysis results and in conjunction with previous research findings on the brain effects of psychotherapy [71], we propose a mechanistic model for the efficacy of CBT, grounded in the neural circuitry of emotions (Figure 3). Initially, patients exhibited reduced activation in the orbital frontal cortex (OFC), which in turn led to diminished PG activation. Post-CBT treatment, the therapy first targeted the OFC, elevating its activation levels. This upregulation subsequently led to increased activity in the PG, restoring it to a normative state. In essence, CBT exerts a top-down effect, initially enhancing OFC activity, which subsequently elevates PG activity. This targeted modulation of the emotional neural circuitry effectively normalizes activation in the relevant brain regions, thereby ameliorating symptoms. Additionally, previous research has demonstrated the significance of the OFC as a key part of the medial network, linking to the cortical circuit that encompasses the PG. This strongly supports our hypothesis [72].

The therapeutic effects of CBT likely extend beyond modulation of specific neural circuits, encompassing broader neurobiological mechanisms, such as neuroplasticity and neurochemical regulation. Research indicates that CBT can induce structural adaptations in key brain regions, including increased gray matter volume in the prefrontal cortex and hippocampus, areas critically involved in cognitive control and memory processes [71]. Concurrently, CBT appears to influence neurotransmitter systems, particularly serotonin and dopamine, which play pivotal roles in mood regulation and reward processing. For instance, reductions in 5-HT1B receptor binding within the dorsal brainstem following CBT have been associated with enhanced emotional regulation [52]. Similarly, CBT may normalize dysregulated dopaminergic activity in the caudate and LN, structures implicated in reward valuation and habitual behaviors [64].

These mechanisms likely operate synergistically: neuroplastic changes may provide the structural foundation for enduring functional reorganization, while neurochemical modulation facilitates rapid adjustments in neural activity. For example, increased OFC activation (as observed in our study) could drive neuroadaptive changes in connected regions, such as the PG, while serotonergic enhancement may optimize the efficiency of this circuitry. This integrative perspective aligns with contemporary frameworks that conceptualize psychotherapy as a targeted “neurobiological intervention” capable of inducing multilevel changes in brain function and structure [25].

Furthermore, interestingly, when we analyzed subgroups according to different anxiety disorder types and task paradigms, we found that patients with specific phobias had a significant increase in left and right SPL activation, as well as a decrease in left and right thalamus activation, after treatment, and this was particularly pronounced in the fear experience task. The primary function of the SPL is to integrate sensory information from different modalities, such as touch, proprioception, and vision, to create a coherent representation of the body in space [73]. This region is also important for spatial attention and perception, as well as for guiding movement and coordinating hand–eye coordination [73]. One study found that individuals with specific phobias showed abnormalities in processing spatial information, such as a possible bias in the perception of spatial distance [74]. This spatial perception abnormality may be associated with reduced activity in the SPL. In addition, patients with specific phobias may have some degree of cognitive bias towards their own location and body representation, which may also be associated with abnormal functioning of the SPL [74].

The thalamus, an important part of the brain located around the ventricles, is a key nucleus of the brain [75]. It is the main transmission station between the cerebral cortex and the hypothalamus, playing an important role in information transfer and integration [76]. The thalamus contains multiple nuclei, each associated with different sensory, motor, emotional, and cognitive functions. In patients with specific phobias, hyperactivation of the thalamus may reflect their hypersensitivity or overreaction to fearful stimuli. This abnormal thalamus activity may be related to aspects of fear memory processing, emotion regulation, and attention allocation [77].

However, the changes in brain activation following treatment for anxiety disorders are less consistent with previous studies. For instance, while some studies have reported increased activation in the caudate and LN following anxiety treatment [78], others have indicated a decrease in activation in these areas [20]. This discrepancy suggests that the neural mechanisms underlying the treatment of anxiety disorders might be more complex and warrant further investigation.

A prior review on the neurobiological alterations following treatment for anxiety disorders, including OCD and PTSD, reported distinct patterns of normalization in neural activity [79]. Specifically, patients with OCD exhibited normalized activity in the orbitofrontal cortex, cingulate cortex, and basal ganglia following CBT. Conversely, patients with PTSD demonstrated adaptive changes predominantly in the temporal lobe and cingulate gyrus. These distinct neural responses to treatment corroborate the diagnostic separation of OCD and PTSD from other anxiety disorders in the Diagnostic and Statistical Manual of Mental Disorders, Fifth Edition (DSM-V), thus indirectly affirming its scientific validity.

In conclusion, our findings reveal more consistent alterations in brain area activation following CBT treatment for anxiety disorders. These results not only contribute to an enhanced understanding of the neurobiological underpinnings of CBT but also offer novel avenues for improving the treatment of anxiety disorders.

### 4.3. Common Changes in Brain Area Activation in CBT for Depressive Disorders and Anxiety Disorders in the Resting State and Task State

In this study, we observed that the brain regions demonstrating alterations post-CBT treatment varied depending on the imaging state employed. Our meta-analysis revealed that during resting-state imaging, heightened activity was predominantly observed in the right and left vACC and the perirhinal cortex. This suggests that CBT primarily targets emotion regulation to alleviate symptoms of depression and anxiety, an effect not observed in task-state imaging. These results resonate with prior studies [22, 23], further substantiating the efficacy of CBT in enhancing cognitive control and emotion regulation mechanisms. Specifically, the right and left ACC are integral to cognitive control, emotion regulation, and decision-making [80]. As such, CBT may ameliorate symptoms by restoring normative levels of activation in these regions, thereby improving cognitive control and emotional regulation. Our findings align with previous research [81], suggesting that CBT alleviates psychological distress by fostering enhanced interaction among neural networks.

Additionally, CBT may also influence how patients process emotional information, thereby modulating the activation patterns in the perirhinal cortex. While traditionally recognized for its role in olfactory processing, the perirhinal cortex also plays a crucial role in emotion processing [82]. Hence, CBT could restore normative activation levels in this region by heightening patients' sensitivity to their emotional states and the emotional information they encounter.

During task-state imaging, our study observed a decrease in activation within the LN and caudate, coupled with an increase in the OFC following CBT for patients with depressive and anxiety disorders. These findings align with prior research [83, 84]. The LN and caudate are critical regions implicated in emotional regulation; their dysregulation has been associated with psychiatric disorders, such as depression and anxiety [85]. Specifically, the caudate is involved in the brain's reward system, an element frequently associated with depressive and anxiety disorders [86]. Conversely, the OFC predominantly serves cognitive control functions, including emotion regulation and decision-making [83]. A previous meta-analysis reported that patients with depressive disorders exhibited deficits in attentional selection and cognitive flexibility in the OFC during task-based exercises [87]. Therefore, the modulatory effects of CBT on these regions likely indicate its beneficial influence on both the emotional and cognitive facets of mental health.

### 4.4. Limitations and Prospects

Our study boasts several strengths. Notably, it employs both ROI and whole-brain analyses, accommodates variations in sample size and statistical thresholds, and executes supplementary analyses to validate the robustness of our findings. We utilized the ALE method, a widely accepted meta-analytic technique, to calculate the probability of voxel-wise activation across different experiments [34]. Furthermore, our work delves into the distinct neurobiological mechanisms of CBT for both anxiety and depressive disorders, offering novel insights into the therapeutic process for each.

However, several limitations should be acknowledged. First, due to the constrained number of available studies, we were unable to analyze the structural images of anxiety and depressive patients after treatment with CBT. Future research incorporating a broader range of studies could bolster the ecological validity of our findings for both anxiety and depressive disorders. Second, selection bias inherent in meta-analysis studies may also have affected our results. Third, the inconsistent reporting of demographic information in the included studies limited our in-depth analysis of the effects of gender and age. Finally, our results are subject to variability stemming from the use of different imaging methodologies and techniques, which could potentially affect the observed impact of CBT [88].

Despite these caveats, our study significantly contributes to the existing literature by characterizing the neural correlates of CBT efficacy in treating anxiety and depressive disorders. The meta-analytic findings lay a strong foundation for future research directions. For instance, our study offers new insights into identifying effective therapeutic targets for CBT, ultimately enhancing its effectiveness for individuals who do not initially respond to treatment. They also offer the opportunity for comparative analyses with other treatment approaches, using neurotherapeutic outcome predictors to gauge efficacy.

Looking forward, there are several avenues for enhancing the rigor and scope of research investigating the effects of CBT on brain activation patterns. Future studies should aim for a more comprehensive and diversified sample selection, standardization of intervention protocols, and enhanced consistency in data sourcing. Additionally, the impact of CBT on neural activation across various demographic groups and clinical conditions merits further exploration. Such investigations could pave the way for CBT to become more effective in treating psychological conditions like anxiety and depressive disorders.

## Figures and Tables

**Table 1:** Characteristics of articles included in meta-analysis.

Study	Disorder	Number	Age (SD)	Females	Sessions (weeks)	Imaging status	Imaging methods	Coordinate space
Burklund et al. [17]	SAD	70	27.74 (7.88)	34	12	Dynamic social threat task	fMRI	MNI
Fu et al. [41]	D	16	40 (9.4)	13	16	Implicit sad faces task	fMRI	Talairach
Furmark et al. [37]	SP	18	35.2 (7.3)	8	9	Resting status	PET	Talairach
Goldapple et al. [18]	D	17	41(9)	11	15–20	Resting status	PET	Talairach
Katayama et al. [38]	D	19	38 (7.4)	10	16	Future-thinking task	fMRI	MNI
Kennedy et al. [16]	D	12	30 (9.8)	7	24	Resting status	PET	MNI
Klumpp et al. [42]	gSAD	21	24.9 (6.3)	15	12	Emotional face task	fMRI	MNI
Klumpp et al. [43]	gSAD	32	25.4 (5.1)	24	12	Emotional face task	fMRI	MNI
Konarski et al. [44]	D	7	32.7 (11.4)	5	12	Resting status	PET	MNI
Lipka et al. [45]	SP	14	25 (3.7)	14	16	Affective stimuli anticipation	fMRI	Talairach
Lueken et al. [46]	PD	49	35.27 (10.43)	33	12	Fear conditioning task	fMRI	MNI
Månsson et al. [40]	SAD	13	32.46 (8.6)	11	4	Emotional face task	fMRI	MNI
Månsson et al. [47]	SAD	26	32.27 (9.7)	22	12	Self-referential criticism task	fMRI	MNI
Paquette et al. [20]	SP	12	24.8 (4.5)	12	9	Fear experience task	fMRI	Talairach
Reinecke et al. [48]	PD	14	37.2 (11.1)	10	12	Emotion regulation task	fMRI	MNI
Ritchey et al. [49]	D	22	36.1 (10.1)	13	9	Emotion evaluation task	fMRI	Talairach
Sakai et al. [50]	PD	12	29.8 (6.2)	9	4	Resting status	PET	Talairach
Sankar et al. [19]	D	16	39.94 (9.48)	13	4	Dysfunctionl attitude scale task	fMRI	Talairach
Schienle et al. [21]	SP	14	27.2 (9.2)	14	24	Fear experience task	fMRI	MNI
Straube et al. [51]	SP	13	21.92 (2.02)	13	16	Fear experience task	fMRI	Talairach
Tiger et al. [52]	D	10	48 (17)	6	NA	Resting status	PET	MNI
Yang et al. [53]	D	16	34.6 (8.29)	14	12	Emotional conflict task	fMRI	MNI

*Note:* The number refers to the number of patients treated with cognitive behavioral therapy. Sessions refer to the mean number of therapy sessions that are usually provided weekly. All values are displayed as mean ± SD unless indicated differently.

Abbreviations: D, depressive disorder; fMRI, functional magnetic resonance imaging; gSAD, generalized social anxiety disorder; MNI, Montreal Neurological Institute; MRI, magnetic resonance imaging; NA, not available; PD, panic disorder; PET, positron emission tomography; SAD, social anxiety disorder; SP, specific phobia.

## Data Availability

The data that support the findings of this study are available from the corresponding author upon reasonable request.

## Supporting Information

Supporting Information See Tables S1–S11 and Figures S1–S3 in the Supporting Information.
