# Supplementary material for: Activation Likelihood Estimation Meta-Analysis of the Effects of Cognitive Behavioral Therapy on Brain Activation in the Treatment of Depression and Anxiety Disorders
Source: Depress Anxiety. 2025 Jun 17;2025:3557367. doi: 10.1155/da/3557367 (PMC12187445; doi:10.1155/da/3557367)
Supplement: Supporting Information — See Tables S1–S11 and Figures S1–S3 in the Supporting Information. [file 3557367.f1.docx]

**Supplementary Table 1. Search strategy for Pubmed**

| Search number | Query | Results |
| --- | --- | --- |
| 17 | ((("Depression"[Mesh]) OR ((((((Depressive Symptoms[Title/Abstract]) OR (Depressive Symptom[Title/Abstract])) OR (Symptom, Depressive[Title/Abstract])) OR (Symptoms, Depressive[Title/Abstract])) OR (Symptoms, Depressive[Title/Abstract])) OR (Depression, Emotional[Title/Abstract]))) AND (("Cognitive Behavioral Therapy"[Mesh]) OR (((((((((((((((((((((((((((((Behavioral Therapies, Cognitive[Title/Abstract]) OR (Behavioral Therapy, Cognitive[Title/Abstract])) OR (Cognitive Behavioral Therapies[Title/Abstract])) OR (Therapies, Cognitive Behavioral[Title/Abstract])) OR (Therapy, Cognitive Behavioral[Title/Abstract])) OR (Behavior Therapy, Cognitive[Title/Abstract])) OR (Cognitive Behavior Therapy[Title/Abstract])) OR (Cognitive Behaviour Therapy[Title/Abstract])) OR (Behaviour Therapies, Cognitive[Title/Abstract])) OR (Behaviour Therapy, Cognitive[Title/Abstract])) OR (Cognitive Behaviour Therapies[Title/Abstract])) OR (Therapies, Cognitive Behaviour[Title/Abstract])) OR (Therapy, Cognitive Behaviour[Title/Abstract])) OR (Cognitive Therapy[Title/Abstract])) OR (Therapy, Cognitive Behavior[Title/Abstract])) OR (Behavior Therapies, Cognitive[Title/Abstract])) OR (Cognitive Behavior Therapies[Title/Abstract])) OR (Therapies, Cognitive Behavior[Title/Abstract])) OR (Cognitive Psychotherapy[Title/Abstract])) OR (Cognitive Psychotherapies[Title/Abstract])) OR (Psychotherapies, Cognitive[Title/Abstract])) OR (Psychotherapy, Cognitive[Title/Abstract])) OR (Therapy, Cognitive[Title/Abstract])) OR (Cognitive Therapies[Title/Abstract])) OR (Therapies, Cognitive[Title/Abstract])) OR (Therapy, Cognition[Title/Abstract])) OR (Cognition Therapy[Title/Abstract])) OR (Cognition Therapies[Title/Abstract])) OR (Therapies, Cognition[Title/Abstract])))) AND ((("Neuroimaging"[Mesh]) OR ((Brain Imaging[Title/Abstract]) OR (Imaging, Brain[Title/Abstract]))) OR (("Magnetic Resonance Imaging"[Mesh]) OR ((((((((((((((((((((((((((((((((((((((((Imaging, Magnetic Resonance[Title/Abstract]) OR (NMR Imaging[Title/Abstract])) OR (Imaging, NMR[Title/Abstract])) OR (Zeugmatography[Title/Abstract])) OR (MR Tomography[Title/Abstract])) OR (NMR Tomography[Title/Abstract])) OR (Tomography, NMR[Title/Abstract])) OR (Steady-State Free Precession MRI[Title/Abstract])) OR (Steady State Free Precession MRI[Title/Abstract])) OR (Tomography, MR[Title/Abstract])) OR (Imaging, Chemical Shift[Title/Abstract])) OR (Chemical Shift Imagings[Title/Abstract])) OR (Imagings, Chemical Shift[Title/Abstract])) OR (Shift Imaging, Chemical[Title/Abstract])) OR (Shift Imagings, Chemical[Title/Abstract])) OR (Chemical Shift Imaging[Title/Abstract])) OR (Magnetic Resonance Image[Title/Abstract])) OR (Image, Magnetic Resonance[Title/Abstract])) OR (Magnetic Resonance Images[Title/Abstract])) OR (Resonance Image, Magnetic[Title/Abstract])) OR (Magnetization Transfer Contrast Imaging[Title/Abstract])) OR (MRI Scans[Title/Abstract])) OR (MRI Scan[Title/Abstract])) OR (Scan, MRI[Title/Abstract])) OR (Scans, MRI[Title/Abstract])) OR (Tomography, Proton Spin[Title/Abstract])) OR (Proton Spin Tomography[Title/Abstract])) OR (fMRI[Title/Abstract])) OR (MRI, Functional[Title/Abstract])) OR (Functional MRI[Title/Abstract])) OR (Functional MRIs[Title/Abstract])) OR (MRIs, Functional[Title/Abstract])) OR (Functional Magnetic Resonance Imaging[Title/Abstract])) OR (Magnetic Resonance Imaging, Functional[Title/Abstract])) OR (Spin Echo Imaging[Title/Abstract])) OR (Echo Imaging, Spin[Title/Abstract])) OR (Echo Imagings, Spin[Title/Abstract])) OR (Imaging, Spin Echo[Title/Abstract])) OR (Imagings, Spin Echo[Title/Abstract])) OR (Spin Echo Imagings[Title/Abstract])))) | 117 |
| 18 | ((("Anxiety"[Mesh]) OR (((((((((((Anxiety Disorder[Title/Abstract]) OR (Disorder, Anxiety[Title/Abstract])) OR (Disorders, Anxiety[Title/Abstract])) OR (Neuroses, Anxiety[Title/Abstract])) OR (Anxiety Neuroses[Title/Abstract])) OR (Anxiety States, Neurotic[Title/Abstract])) OR (Anxiety State, Neurotic[Title/Abstract])) OR (Neurotic Anxiety State[Title/Abstract])) OR (Neurotic Anxiety States[Title/Abstract])) OR (State, Neurotic Anxiety[Title/Abstract])) OR (States, Neurotic Anxiety[Title/Abstract]))) AND (("Cognitive Behavioral Therapy"[Mesh]) OR (((((((((((((((((((((((((((((Behavioral Therapies, Cognitive[Title/Abstract]) OR (Behavioral Therapy, Cognitive[Title/Abstract])) OR (Cognitive Behavioral Therapies[Title/Abstract])) OR (Therapies, Cognitive Behavioral[Title/Abstract])) OR (Therapy, Cognitive Behavioral[Title/Abstract])) OR (Behavior Therapy, Cognitive[Title/Abstract])) OR (Cognitive Behavior Therapy[Title/Abstract])) OR (Cognitive Behaviour Therapy[Title/Abstract])) OR (Behaviour Therapies, Cognitive[Title/Abstract])) OR (Behaviour Therapy, Cognitive[Title/Abstract])) OR (Cognitive Behaviour Therapies[Title/Abstract])) OR (Therapies, Cognitive Behaviour[Title/Abstract])) OR (Therapy, Cognitive Behaviour[Title/Abstract])) OR (Cognitive Therapy[Title/Abstract])) OR (Therapy, Cognitive Behavior[Title/Abstract])) OR (Behavior Therapies, Cognitive[Title/Abstract])) OR (Cognitive Behavior Therapies[Title/Abstract])) OR (Therapies, Cognitive Behavior[Title/Abstract])) OR (Cognitive Psychotherapy[Title/Abstract])) OR (Cognitive Psychotherapies[Title/Abstract])) OR (Psychotherapies, Cognitive[Title/Abstract])) OR (Psychotherapy, Cognitive[Title/Abstract])) OR (Therapy, Cognitive[Title/Abstract])) OR (Cognitive Therapies[Title/Abstract])) OR (Therapies, Cognitive[Title/Abstract])) OR (Therapy, Cognition[Title/Abstract])) OR (Cognition Therapy[Title/Abstract])) OR (Cognition Therapies[Title/Abstract])) OR (Therapies, Cognition[Title/Abstract])))) AND ((("Neuroimaging"[Mesh]) OR ((Brain Imaging[Title/Abstract]) OR (Imaging, Brain[Title/Abstract]))) OR (("Magnetic Resonance Imaging"[Mesh]) OR ((((((((((((((((((((((((((((((((((((((((Imaging, Magnetic Resonance[Title/Abstract]) OR (NMR Imaging[Title/Abstract])) OR (Imaging, NMR[Title/Abstract])) OR (Zeugmatography[Title/Abstract])) OR (MR Tomography[Title/Abstract])) OR (NMR Tomography[Title/Abstract])) OR (Tomography, NMR[Title/Abstract])) OR (Steady-State Free Precession MRI[Title/Abstract])) OR (Steady State Free Precession MRI[Title/Abstract])) OR (Tomography, MR[Title/Abstract])) OR (Imaging, Chemical Shift[Title/Abstract])) OR (Chemical Shift Imagings[Title/Abstract])) OR (Imagings, Chemical Shift[Title/Abstract])) OR (Shift Imaging, Chemical[Title/Abstract])) OR (Shift Imagings, Chemical[Title/Abstract])) OR (Chemical Shift Imaging[Title/Abstract])) OR (Magnetic Resonance Image[Title/Abstract])) OR (Image, Magnetic Resonance[Title/Abstract])) OR (Magnetic Resonance Images[Title/Abstract])) OR (Resonance Image, Magnetic[Title/Abstract])) OR (Magnetization Transfer Contrast Imaging[Title/Abstract])) OR (MRI Scans[Title/Abstract])) OR (MRI Scan[Title/Abstract])) OR (Scan, MRI[Title/Abstract])) OR (Scans, MRI[Title/Abstract])) OR (Tomography, Proton Spin[Title/Abstract])) OR (Proton Spin Tomography[Title/Abstract])) OR (fMRI[Title/Abstract])) OR (MRI, Functional[Title/Abstract])) OR (Functional MRI[Title/Abstract])) OR (Functional MRIs[Title/Abstract])) OR (MRIs, Functional[Title/Abstract])) OR (Functional Magnetic Resonance Imaging[Title/Abstract])) OR (Magnetic Resonance Imaging, Functional[Title/Abstract])) OR (Spin Echo Imaging[Title/Abstract])) OR (Echo Imaging, Spin[Title/Abstract])) OR (Echo Imagings, Spin[Title/Abstract])) OR (Imaging, Spin Echo[Title/Abstract])) OR (Imagings, Spin Echo[Title/Abstract])) OR (Spin Echo Imagings[Title/Abstract])))) | 114 |
| 16 | (("Neuroimaging"[Mesh]) OR ((Brain Imaging[Title/Abstract]) OR (Imaging, Brain[Title/Abstract]))) OR (("Magnetic Resonance Imaging"[Mesh]) OR ((((((((((((((((((((((((((((((((((((((((Imaging, Magnetic Resonance[Title/Abstract]) OR (NMR Imaging[Title/Abstract])) OR (Imaging, NMR[Title/Abstract])) OR (Zeugmatography[Title/Abstract])) OR (MR Tomography[Title/Abstract])) OR (NMR Tomography[Title/Abstract])) OR (Tomography, NMR[Title/Abstract])) OR (Steady-State Free Precession MRI[Title/Abstract])) OR (Steady State Free Precession MRI[Title/Abstract])) OR (Tomography, MR[Title/Abstract])) OR (Imaging, Chemical Shift[Title/Abstract])) OR (Chemical Shift Imagings[Title/Abstract])) OR (Imagings, Chemical Shift[Title/Abstract])) OR (Shift Imaging, Chemical[Title/Abstract])) OR (Shift Imagings, Chemical[Title/Abstract])) OR (Chemical Shift Imaging[Title/Abstract])) OR (Magnetic Resonance Image[Title/Abstract])) OR (Image, Magnetic Resonance[Title/Abstract])) OR (Magnetic Resonance Images[Title/Abstract])) OR (Resonance Image, Magnetic[Title/Abstract])) OR (Magnetization Transfer Contrast Imaging[Title/Abstract])) OR (MRI Scans[Title/Abstract])) OR (MRI Scan[Title/Abstract])) OR (Scan, MRI[Title/Abstract])) OR (Scans, MRI[Title/Abstract])) OR (Tomography, Proton Spin[Title/Abstract])) OR (Proton Spin Tomography[Title/Abstract])) OR (fMRI[Title/Abstract])) OR (MRI, Functional[Title/Abstract])) OR (Functional MRI[Title/Abstract])) OR (Functional MRIs[Title/Abstract])) OR (MRIs, Functional[Title/Abstract])) OR (Functional Magnetic Resonance Imaging[Title/Abstract])) OR (Magnetic Resonance Imaging, Functional[Title/Abstract])) OR (Spin Echo Imaging[Title/Abstract])) OR (Echo Imaging, Spin[Title/Abstract])) OR (Echo Imagings, Spin[Title/Abstract])) OR (Imaging, Spin Echo[Title/Abstract])) OR (Imagings, Spin Echo[Title/Abstract])) OR (Spin Echo Imagings[Title/Abstract]))) | 669,375 |
| 15 | ("Magnetic Resonance Imaging"[Mesh]) OR ((((((((((((((((((((((((((((((((((((((((Imaging, Magnetic Resonance[Title/Abstract]) OR (NMR Imaging[Title/Abstract])) OR (Imaging, NMR[Title/Abstract])) OR (Zeugmatography[Title/Abstract])) OR (MR Tomography[Title/Abstract])) OR (NMR Tomography[Title/Abstract])) OR (Tomography, NMR[Title/Abstract])) OR (Steady-State Free Precession MRI[Title/Abstract])) OR (Steady State Free Precession MRI[Title/Abstract])) OR (Tomography, MR[Title/Abstract])) OR (Imaging, Chemical Shift[Title/Abstract])) OR (Chemical Shift Imagings[Title/Abstract])) OR (Imagings, Chemical Shift[Title/Abstract])) OR (Shift Imaging, Chemical[Title/Abstract])) OR (Shift Imagings, Chemical[Title/Abstract])) OR (Chemical Shift Imaging[Title/Abstract])) OR (Magnetic Resonance Image[Title/Abstract])) OR (Image, Magnetic Resonance[Title/Abstract])) OR (Magnetic Resonance Images[Title/Abstract])) OR (Resonance Image, Magnetic[Title/Abstract])) OR (Magnetization Transfer Contrast Imaging[Title/Abstract])) OR (MRI Scans[Title/Abstract])) OR (MRI Scan[Title/Abstract])) OR (Scan, MRI[Title/Abstract])) OR (Scans, MRI[Title/Abstract])) OR (Tomography, Proton Spin[Title/Abstract])) OR (Proton Spin Tomography[Title/Abstract])) OR (fMRI[Title/Abstract])) OR (MRI, Functional[Title/Abstract])) OR (Functional MRI[Title/Abstract])) OR (Functional MRIs[Title/Abstract])) OR (MRIs, Functional[Title/Abstract])) OR (Functional Magnetic Resonance Imaging[Title/Abstract])) OR (Magnetic Resonance Imaging, Functional[Title/Abstract])) OR (Spin Echo Imaging[Title/Abstract])) OR (Echo Imaging, Spin[Title/Abstract])) OR (Echo Imagings, Spin[Title/Abstract])) OR (Imaging, Spin Echo[Title/Abstract])) OR (Imagings, Spin Echo[Title/Abstract])) OR (Spin Echo Imagings[Title/Abstract])) | 550,606 |
| 14 | (((((((((((((((((((((((((((((((((((((((Imaging, Magnetic Resonance[Title/Abstract]) OR (NMR Imaging[Title/Abstract])) OR (Imaging, NMR[Title/Abstract])) OR (Zeugmatography[Title/Abstract])) OR (MR Tomography[Title/Abstract])) OR (NMR Tomography[Title/Abstract])) OR (Tomography, NMR[Title/Abstract])) OR (Steady-State Free Precession MRI[Title/Abstract])) OR (Steady State Free Precession MRI[Title/Abstract])) OR (Tomography, MR[Title/Abstract])) OR (Imaging, Chemical Shift[Title/Abstract])) OR (Chemical Shift Imagings[Title/Abstract])) OR (Imagings, Chemical Shift[Title/Abstract])) OR (Shift Imaging, Chemical[Title/Abstract])) OR (Shift Imagings, Chemical[Title/Abstract])) OR (Chemical Shift Imaging[Title/Abstract])) OR (Magnetic Resonance Image[Title/Abstract])) OR (Image, Magnetic Resonance[Title/Abstract])) OR (Magnetic Resonance Images[Title/Abstract])) OR (Resonance Image, Magnetic[Title/Abstract])) OR (Magnetization Transfer Contrast Imaging[Title/Abstract])) OR (MRI Scans[Title/Abstract])) OR (MRI Scan[Title/Abstract])) OR (Scan, MRI[Title/Abstract])) OR (Scans, MRI[Title/Abstract])) OR (Tomography, Proton Spin[Title/Abstract])) OR (Proton Spin Tomography[Title/Abstract])) OR (fMRI[Title/Abstract])) OR (MRI, Functional[Title/Abstract])) OR (Functional MRI[Title/Abstract])) OR (Functional MRIs[Title/Abstract])) OR (MRIs, Functional[Title/Abstract])) OR (Functional Magnetic Resonance Imaging[Title/Abstract])) OR (Magnetic Resonance Imaging, Functional[Title/Abstract])) OR (Spin Echo Imaging[Title/Abstract])) OR (Echo Imaging, Spin[Title/Abstract])) OR (Echo Imagings, Spin[Title/Abstract])) OR (Imaging, Spin Echo[Title/Abstract])) OR (Imagings, Spin Echo[Title/Abstract])) OR (Spin Echo Imagings[Title/Abstract]) | 123,772 |
| 13 | "Magnetic Resonance Imaging"[Mesh] | 517,469 |
| 12 | ("Neuroimaging"[Mesh]) OR ((Brain Imaging[Title/Abstract]) OR (Imaging, Brain[Title/Abstract])) | 204,640 |
| 11 | (Brain Imaging[Title/Abstract]) OR (Imaging, Brain[Title/Abstract]) | 17,416 |
| 10 | "Neuroimaging"[Mesh] | 191,473 |
| 9 | ("Cognitive Behavioral Therapy"[Mesh]) OR (((((((((((((((((((((((((((((Behavioral Therapies, Cognitive[Title/Abstract]) OR (Behavioral Therapy, Cognitive[Title/Abstract])) OR (Cognitive Behavioral Therapies[Title/Abstract])) OR (Therapies, Cognitive Behavioral[Title/Abstract])) OR (Therapy, Cognitive Behavioral[Title/Abstract])) OR (Behavior Therapy, Cognitive[Title/Abstract])) OR (Cognitive Behavior Therapy[Title/Abstract])) OR (Cognitive Behaviour Therapy[Title/Abstract])) OR (Behaviour Therapies, Cognitive[Title/Abstract])) OR (Behaviour Therapy, Cognitive[Title/Abstract])) OR (Cognitive Behaviour Therapies[Title/Abstract])) OR (Therapies, Cognitive Behaviour[Title/Abstract])) OR (Therapy, Cognitive Behaviour[Title/Abstract])) OR (Cognitive Therapy[Title/Abstract])) OR (Therapy, Cognitive Behavior[Title/Abstract])) OR (Behavior Therapies, Cognitive[Title/Abstract])) OR (Cognitive Behavior Therapies[Title/Abstract])) OR (Therapies, Cognitive Behavior[Title/Abstract])) OR (Cognitive Psychotherapy[Title/Abstract])) OR (Cognitive Psychotherapies[Title/Abstract])) OR (Psychotherapies, Cognitive[Title/Abstract])) OR (Psychotherapy, Cognitive[Title/Abstract])) OR (Therapy, Cognitive[Title/Abstract])) OR (Cognitive Therapies[Title/Abstract])) OR (Therapies, Cognitive[Title/Abstract])) OR (Therapy, Cognition[Title/Abstract])) OR (Cognition Therapy[Title/Abstract])) OR (Cognition Therapies[Title/Abstract])) OR (Therapies, Cognition[Title/Abstract])) | 66,453 |
| 8 | ((((((((((((((((((((((((((((Behavioral Therapies, Cognitive[Title/Abstract]) OR (Behavioral Therapy, Cognitive[Title/Abstract])) OR (Cognitive Behavioral Therapies[Title/Abstract])) OR (Therapies, Cognitive Behavioral[Title/Abstract])) OR (Therapy, Cognitive Behavioral[Title/Abstract])) OR (Behavior Therapy, Cognitive[Title/Abstract])) OR (Cognitive Behavior Therapy[Title/Abstract])) OR (Cognitive Behaviour Therapy[Title/Abstract])) OR (Behaviour Therapies, Cognitive[Title/Abstract])) OR (Behaviour Therapy, Cognitive[Title/Abstract])) OR (Cognitive Behaviour Therapies[Title/Abstract])) OR (Therapies, Cognitive Behaviour[Title/Abstract])) OR (Therapy, Cognitive Behaviour[Title/Abstract])) OR (Cognitive Therapy[Title/Abstract])) OR (Therapy, Cognitive Behavior[Title/Abstract])) OR (Behavior Therapies, Cognitive[Title/Abstract])) OR (Cognitive Behavior Therapies[Title/Abstract])) OR (Therapies, Cognitive Behavior[Title/Abstract])) OR (Cognitive Psychotherapy[Title/Abstract])) OR (Cognitive Psychotherapies[Title/Abstract])) OR (Psychotherapies, Cognitive[Title/Abstract])) OR (Psychotherapy, Cognitive[Title/Abstract])) OR (Therapy, Cognitive[Title/Abstract])) OR (Cognitive Therapies[Title/Abstract])) OR (Therapies, Cognitive[Title/Abstract])) OR (Therapy, Cognition[Title/Abstract])) OR (Cognition Therapy[Title/Abstract])) OR (Cognition Therapies[Title/Abstract])) OR (Therapies, Cognition[Title/Abstract]) | 38,349 |
| 7 | "Cognitive Behavioral Therapy"[Mesh] | 34,774 |
| 6 | ("Anxiety"[Mesh]) OR (((((((((((Anxiety Disorder[Title/Abstract]) OR (Disorder, Anxiety[Title/Abstract])) OR (Disorders, Anxiety[Title/Abstract])) OR (Neuroses, Anxiety[Title/Abstract])) OR (Anxiety Neuroses[Title/Abstract])) OR (Anxiety States, Neurotic[Title/Abstract])) OR (Anxiety State, Neurotic[Title/Abstract])) OR (Neurotic Anxiety State[Title/Abstract])) OR (Neurotic Anxiety States[Title/Abstract])) OR (State, Neurotic Anxiety[Title/Abstract])) OR (States, Neurotic Anxiety[Title/Abstract])) | 122,854 |
| 5 | ((((((((((Anxiety Disorder[Title/Abstract]) OR (Disorder, Anxiety[Title/Abstract])) OR (Disorders, Anxiety[Title/Abstract])) OR (Neuroses, Anxiety[Title/Abstract])) OR (Anxiety Neuroses[Title/Abstract])) OR (Anxiety States, Neurotic[Title/Abstract])) OR (Anxiety State, Neurotic[Title/Abstract])) OR (Neurotic Anxiety State[Title/Abstract])) OR (Neurotic Anxiety States[Title/Abstract])) OR (State, Neurotic Anxiety[Title/Abstract])) OR (States, Neurotic Anxiety[Title/Abstract]) | 21,858 |
| 4 | "Anxiety"[Mesh] | 105,255 |
| 3 | ("Depression"[Mesh]) OR ((((((Depressive Symptoms[Title/Abstract]) OR (Depressive Symptom[Title/Abstract])) OR (Symptom, Depressive[Title/Abstract])) OR (Symptoms, Depressive[Title/Abstract])) OR (Symptoms, Depressive[Title/Abstract])) OR (Depression, Emotional[Title/Abstract])) | 175,767 |
| 2 | (((((Depressive Symptoms[Title/Abstract]) OR (Depressive Symptom[Title/Abstract])) OR (Symptom, Depressive[Title/Abstract])) OR (Symptoms, Depressive[Title/Abstract])) OR (Symptoms, Depressive[Title/Abstract])) OR (Depression, Emotional[Title/Abstract]) | 61,878 |
| 1 | "Depression"[Mesh] | 143,597 |

**Supplementary Table 2. Quality assessment checklist (total score out of 10)**

| **Criteria 1: Participants** |
| --- |
| 1. Patients were prospectively assessed; specific diagnostic criteria and demographic data were reported. |
| 2. Healthy controls were prospectively evaluated; psychiatric and medical illnesses were excluded. |
| 3. Important variables (e.g., age, sex, illness duration, onset, medication status, comorbidity, and the severity of illness) were reported. |
| 4. Sample size per group $\geq$ 10. |
| **Criteria 2: Methods for image acquisition and analysis** |
| 5. Whole-brain analysis was automated with no a priori regional selection. |
| 6. Coordinates reported in a standard space. |
| 7. The imaging technique used was clearly described so that it could be reproduced. |
| 8. Measurements were clearly described so that they could be reproduced. |
| **Criteria 3: Results and conclusions** |
| 9. Statistical parameters for significant nonsignificant differences were provided. |
| 10. Conclusions were consistent with the results obtained and the limitations were discussed. |
| Didn’t match the criteria, 0 points were awarded; |
| Partially met the criteria, 0.5 points were awarded; |
| Fully met the criteria, 1 point was awarded. |

**Supplementary Table 3. The CBT treatment effects of the included articles**

| **Study** | **Result** |
| --- | --- |
| Burklund et al. (2017) | The SAD group evidenced a different pattern, with no differences in these rejection regions and relatively greater activations in the amygdala and other regions to neutral stimuli. Greater responses in both rejection regions and the amygdala to rejection vs.neutral stimuli **predicted better CBT outcomes.** |
| Fu et al. (2008) | Thirteen patients (81.2%) met a 50% decrease in HRSD criteria for a full clinical response, although only 9 (56.3%) of the 16 patients met criteria for full remission **with a final HRSD ≤ 7. (Before CBT: Mean HRSD Score 20.9)** |
| Furmark et al. (2002) | **Symptoms improved significantly** and roughly equally with citalopram and cognitive-behavioral therapy, whereas the waiting-list group remained unchanged. |
| Goldapple et al. (2004) | A full course of **CBT resulted in significant clinical improvement** in the 14 study completers (mean±SD posttreatment Hamilton Depression Rating Scale score of 6.7±4). |
| Katayama et al. (2022) | **Greater alleviation of depressive symptoms was observed in the CBT**—compared to the TC—group at the 6-month follow-up assessment (−5.9, 95% CI: −11.7 to −0.05, p = 0.048; Table A3). |
| Kennedy et al. (2007) | In the CBT group, the **mean HAMD scores were 20.6 (SD = 3.4) at baseline and 9.8 (SD = 7.6) at endpoint,** with a mean drop in severity of 53% (t= 4.83, df = 11, p = 0.001). |
| Klumpp et al. (2014) | After 12 weeks of individual CBT, gSAD symptom severity, as assessed with the LSAS, **significantly decreased from an average of 72.5 (S.D. =11.6) to 50.4 (S.D. =19.5) (t =5.51, p<0.001).** |
| Klump et al. (2016) | After completing all 12 weeks of CBT, gSAD symptom severity, as assessed with LSAS, **significantly decreased from an average of 74.3 1**±**4.9 to 47.9**±**20.4 [(31) t = 7.1, P<0.001].** |
| Konarski et al. (2009) | In the CBT group, the **mean HAMD scores were 19.6 (3.5) at baseline and 5.4 (3.8) at endpoint.** |
| Lipka et al. (2014) | **Successful CBT was reflected in an overall downregulation in these fear circuitry structures**, especially in the right amygdala and anterior cingulate cortex, with reductions in amygdala responsiveness associated with **self-reported symptom improvement.** |
| Lueken et al. (2013) | In the CBT group, the **mean Anxiety Sensitivity Index total scores were 32.56 (8.28) at baseline and 13.24 (7.81) at endpoint.** |
| Månsson et al. (2013) | Moderate to large within-group effect sizes (Cohen's d) were observed on LSAS-SR, d = 0.91 (CI 95% 0.44–1.37) for iCBT, and d = 1.08 (CI 95% 0.43–1.74) for ABM. A repeated measure MANOVA including all social anxiety self-reports (LSAS-SR, SIAS, SPS and SPSQ) revealed a significant multivariate time group interaction effect, Wilks's λ = 0.535, F (4,19) = 4.13, p = 0.01, suggesting **a differential treatment outcome in favor of iCBT.** |
| Månsson et al. (2016) | The clinician administrated CGI-I assessments **revealed significantly more participants responding positively to the CBT** (61%, 8/13) than to the ABM control treatment (23%, 3/13; χ2 = 3.94, P= 0.047), and on the self-report questionnaires, we found similar results in favor of CBT. |
| Paquette et al. (2003) | The post-CBT average subjective rating **was significantly lower than** that observed before CBT (P < 0.001). |
| Reinecke et al. (2014) | After having received treatment, **patients showed significant improvement compared** to their baseline scores in trait anxiety and depression (HADS) and panic severity (PDSS-SR). |
| Ritchey et al.(2011) | There was also a marginal interaction between emotion type and session, F(2, 10) =3.33, p = 0.08, reflecting a **modest shift toward higher ratings for the positive pictures after treatment.** |
| Sakai et al.(2006) | The PDSS, its first and second subscales, the number of panic attacks during the previous 4 weeks, the state anxiety subscale of STAI, and the HAM-D **were significantly improved by the treatment** in 11 patients |
| Sankar et al. (2015) | The extreme responses to the DAS statements showed a significant group × time interaction effect (F1,30 = 7.434, p = 0.011), in which patients showed a **significant reduction in mean number of extreme responses following a course of CBT** [t = 2.938, degrees of freedom (df) = 15, p = 0.010]. |
| Schienle et al. (2007) | The therapy group (TG) reported **a pronounced reduction in phobic symptoms (SPQ) from the first to the second session**, whereas the SPQ scores for the waiting list group (WG) hardly changed. The conducted analysis of variance with the factors group (WG, TG) and session (1, 2) resulted in a significant interaction effect (F(1,24) = 165, P < 0.001). |
| Straube et al. (2006) | **CBT strongly reduced phobic symptoms** in the TG while the WG remained behaviorally unchanged |
| Tiger et al. (2014) | The patients were improved also based on the CGI with average CGI score for disease severity (CGI-S) **going down from 3.7 to 1.5 (p < 0.001)**. |
| Yang et al. (2018) | After twelve weeks, RT was **significantly decreased in patients following CBT** [t(30) = 2.75, p = 0.01, d = 0.51], but not in healthy controls (p > 0.20). After treatment, patients’ depressive symptoms assessed by MADRS were **also significantly improved** [t(30) = -6.15, p < 0.001, d = 1.12; mean reduction relative to baseline: 66.31%]. |

**Supplementary Table 4. ALE meta-analysis activation cluster results for MDD in resting status**

| Hemisphere | BA | Effect lobe | Effect gyrus | Center coordinates | | | Volume/mm³ | ALE value* |
| --- | --- | --- | --- | --- | --- | --- | --- | --- |
|  |  |  |  | X | Y | Z |  |  |
| Increase: |  |  |  |  |  |  |  |  |
| left | 24 | Limbic Lobe | Ventral anterior cingulate cortex | -8 | 6 | 28 | 288 | 0.726 |
| left | 4 | Limbic Lobe | Parahippocampal Gyrus | -28 | -38 | -10 | 272 | 0.723 |
| right | 24 | Limbic Lobe | Ventral anterior cingulate cortex | 16 | -14 | 34 | 256 | 0.735 |
| right | 21 | Temporal Lobe | Sub-Gyral | 42 | -10 | -20 | 224 | 0.750 |
| left | 20 | Temporal Lobe | Inferior Temporal Gyrus | -60 | -38 | -16 | 216 | 0.714 |
| right | 19 | Occipital Lobe | Inferior Occipital Gyrus | 52 | -74 | -6 | 200 | 0.714 |

*The results are in scientific notation and are actually ALE value x 10^-2^. BA: Brodmann Area. The peak coordinates were in the MNI system. Clusters identified in the meta-analysis were obtained after controlling the false discovery rate corrected at P<0.001 and applying a minimum cluster size of 200 mm3. ALE: Activation likelihood estimation; BA: Brodmann Area.

**Supplementary Table 5.** **ALE meta-analysis activation cluster results for AD in resting status**

| Hemisphere | BA | Effect lobe | Effect gyrus | Center coordinates | | | Volume/mm³ | ALE value* |
| --- | --- | --- | --- | --- | --- | --- | --- | --- |
|  |  |  |  | X | Y | Z |  |  |
| Increase: |  |  |  |  |  |  |  |  |
| right | 10 | Frontal Lobe | Superior Frontal Gyrus | 16 | 66 | 4 | 936 | 0.686 |
| left | 9 | Frontal Lobe | Superior Frontal Gyrus | -8 | 64 | 22 | 888 | 0.671 |
| decrease: |  |  |  |  |  |  |  |  |
| right | Hippocampus | Temporal Lobe | Caudate | 36 | -34 | -4 | 240 | 0.697 |

*The results are in scientific notation and are actually ALE value x 10^-2^. BA: Brodmann Area. The peak coordinates were in the MNI system. Clusters identified in the meta-analysis were obtained after controlling the false discovery rate corrected at P<0.001 and applying a minimum cluster size of 200 mm3. ALE: Activation likelihood estimation; BA: Brodmann Area.

**Supplementary Table 6.** **Common changes in brain area activation in CBT for depressive disorders and anxiety disorders in the resting state and task state**

| Imaging Status | Hemisphere | BA | Effect lobe | Effect gyrus | Center coordinates | | | Volume/mm³ | ALE value* |
| --- | --- | --- | --- | --- | --- | --- | --- | --- | --- |
|  |  |  |  |  | X | Y | Z |  |  |
| Resting Status | Increase: |  |  |  |  |  |  |  |  |
|  | right | 24 | Limbic Lobe | Ventral anterior cingulate cortex | 16 | -14 | 34 | 216 | 0.735 |
|  | left | 36 | Limbic Lobe | Perirhinal cortex | -28 | -38 | -10 | 208 | 0.722 |
|  | left | 24 | Limbic Lobe | Ventral anterior cingulate cortex | -8 | 6 | 28 | 200 | 0.725 |
| Task Status | Increase: |  |  |  |  |  |  |  |  |
|  | right | 11 | Frontal Lobe | Orbital frontal lobe | 4 | 46 | -24 | 360 | 0.946 |
|  | Decrease: |  |  |  |  |  |  |  |  |
|  | left | Lateral Globus Pallidus | Sub-lobar | Lentiform Nucleus | -16 | 2 | -14 | 216 | 1.192 |
|  | right | Caudate Body | Sub-lobar | Caudate | 12 | 8 | 12 | 208 | 1.068 |

*The results are in scientific notation and are actually ALE value x 10^-2^. BA: Brodmann Area. The peak coordinates were in the MNI system. Clusters identified in the meta-analysis were obtained after controlling the false discovery rate corrected at P<0.001 and applying a minimum cluster size of 200 mm3. ALE: Activation likelihood estimation; BA: Brodmann Area.

**Supplementary Table 7.** **ALE meta-analysis activation cluster results for AD in task status**

| Hemisphere | BA | Effect lobe | Effect gyrus | Center coordinates | | | Volume/mm³ | ALE value* |
| --- | --- | --- | --- | --- | --- | --- | --- | --- |
|  |  |  |  | X | Y | Z |  |  |
| Increase: |  |  |  |  |  |  |  |  |
| right | 11 | Frontal Lobe | Orbital frontal lobe | 4 | 46 | -24 | 432 | 0.739 |
| right | Hippocampus | Limbic Lobe | Parahippocampal Gyrus | 40 | -18 | -18 | 216 | 0886 |
| right | 32 | Limbic Lobe | Dorsal anterior cingulate cortex | 8 | 36 | -14 | 216 | 0.851 |
| decrease: |  |  |  |  |  |  |  |  |
| left | Lateral Globus Pallidus | Sub-lobar | Lentiform Nucleus | -16 | 2 | -14 | 272 | 1.192 |
| right | Caudate Body | Sub-lobar | Caudate | 12 | 8 | 12 | 248 | 1.068 |
| left | 34 | Limbic Lobe | Dorsal entorhinal cortex | -28 | 2 | -22 | 208 | 1.016 |

*The results are in scientific notation and are actually ALE value x 10^-2^. BA: Brodmann Area. The peak coordinates were in the MNI system. Clusters identified in the meta-analysis were obtained after controlling the false discovery rate corrected at P<0.001 and applying a minimum cluster size of 200 mm3. ALE: Activation likelihood estimation; BA: Brodmann Area.

**Supplementary Table 8. ALE meta-analysis activation cluster results for Different types of anxiety disorders**

| Disease Type | Hemisphere | BA | Effect lobe | Effect gyrus | Center coordinates | | | Volume/mm³ | ALE value* |
| --- | --- | --- | --- | --- | --- | --- | --- | --- | --- |
|  |  |  |  |  | X | Y | Z |  |  |
| Social anxiety disorder | Decrease: |  |  |  |  |  |  |  |  |
|  | left | Lateral Globus Pallidus | Sub-lobar | Lentiform Nucleus | -16 | 2 | -14 | 392 | 1.188 |
| Specific Phobia | Increase: |  |  |  |  |  |  |  |  |
|  | right | 11 | Frontal Lobe | Orbital frontal lobe | 4 | 45 | -22 | 232 | 0.698 |
|  | right | 7 | Parietal Lobe | Superior Parietal Lobule | 34 | -50 | 64 | 232 | 0.678 |
|  | left | 7 | Parietal Lobe | Superior Parietal Lobule | -18 | -54 | 70 | 232 | 0.697 |
|  | right | 9 | Frontal Lobe | Inferior Frontal Gyrus | 46 | 14 | 20 | 224 | 0.692 |
|  | right | 32 | Limbic Lobe | Dorsal anterior cingulate cortex | 26 | 18 | 30 | 224 | 0.733 |

*The results are in scientific notation and are actually ALE value x 10^-2^. BA: Brodmann Area. The peak coordinates were in the MNI system. Clusters identified in the meta-analysis were obtained after controlling the false discovery rate corrected at P<0.001 and applying a minimum cluster size of 200 mm3. ALE: Activation likelihood estimation; BA: Brodmann Area.

**Supplementary Table 9.** **ALE meta-analysis activation cluster results for Emotional face task**

| Hemisphere | BA | Effect lobe | Effect gyrus | Center coordinates | | | Volume/mm³ | ALE value* |
| --- | --- | --- | --- | --- | --- | --- | --- | --- |
|  |  |  |  | X | Y | Z |  |  |
| Increase: |  |  |  |  |  |  |  |  |
| right | 32 | Limbic Lobe | Dorsal anterior cingulate cortex | 8 | 36 | -14 | 1432 | 0.851 |
| left | 4 | Limbic Lobe | Paracentral Lobule | -6 | -34 | 78 | 392 | 0.704 |

*The results are in scientific notation and are actually ALE value x 10^-2^. BA: Brodmann Area. The peak coordinates were in the MNI system. Clusters identified in the meta-analysis were obtained after controlling the false discovery rate corrected at P<0.001 and applying a minimum cluster size of 200 mm3. ALE: Activation likelihood estimation; BA: Brodmann Area.

**Supplementary Table 10.** **ALE meta-analysis activation cluster results for Fear experience task**

| Hemisphere | BA | Effect lobe | Effect gyrus | Center coordinates | | | Volume/mm³ | ALE value* |
| --- | --- | --- | --- | --- | --- | --- | --- | --- |
|  |  |  |  | X | Y | Z |  |  |
| Increase: |  |  |  |  |  |  |  |  |
| right | 18 | Occipital Lobe | Middle occipital gyrus | 40 | -86 | 6 | 232 | 0.698 |
| right | 7 | Parietal Lobe | Superior parietal lobule | 34 | -50 | 64 | 232 | 0.678 |
| right | 7 | Parietal Lobe | Superior parietal lobule | -18 | -54 | 70 | 232 | 0.697 |
| right | 9 | Frontal Lobe | Inferior Frontal Gyrus | 46 | 14 | 20 | 224 | 0.692 |
| right | 32 | Limbic Lobe | Dorsal anterior cingulate cortex | 26 | 18 | 30 | 224 | 0.733 |
| decrease: |  |  |  |  |  |  |  |  |
| leftt | 34 | Limbic Lobe | Dorsal entorhinal cortex | -32 | 6 | -20 | 224 | 0.712 |
| right | Ventral Lateral Nucleus | Sub-lobar | Thalamus | 20 | -12 | 8 | 208 | 0.723 |
| left | Medial Dorsal Nucleus | Sub-lobar | Thalamus | -8 | -12 | 9 | 208 | 0.698 |
| right | 13 | Sub-lobar | Insula | 42 | 6 | -8 | 216 | 0.733 |

*The results are in scientific notation and are actually ALE value x 10^-2^. BA: Brodmann Area. The peak coordinates were in the MNI system. Clusters identified in the meta-analysis were obtained after controlling the false discovery rate corrected at P<0.001 and applying a minimum cluster size of 200 mm3. ALE: Activation likelihood estimation; BA: Brodmann Area

| **Supplementary Table 11.** Quality assessment of included studies | | | | |
| --- | --- | --- | --- | --- |
| Study ID | Criteria 1 | Criteria 2 | Criteria 3 | Quality |
|  | (Max 4 points) | (Max 4 points) | (Max 2 points) |  |
| Burklund et al. (2017) | 4 | 4 | 2 | 10 |
| Fu et al. (2008) | 4 | 3 | 2 | 9 |
| Furmark et al. (2002) | 3.5 | 4 | 2 | 9.5 |
| Goldapple et al. (2004) | 4 | 4 | 2 | 10 |
| Katayama et al. (2022) | 4 | 3 | 2 | 9 |
| Kennedy et al. (2007) | 4 | 3 | 1.3 | 9.5 |
| Klumpp et al. (2014) | 4 | 4 | 2 | 10 |
| Klump et al. (2016) | 4 | 4 | 2 | 10 |
| Konarski et al. (2009) | 3 | 4 | 2 | 9 |
| Lipka et al. (2014) | 4 | 3.5 | 2 | 9.5 |
| Lueken et al. (2013) | 3.5 | 4 | 2 | 9.5 |
| Månsson et al. (2013) | 4 | 4 | 2 | 10 |
| Månsson et al. (2016) | 4 | 4 | 2 | 10 |
| Paquette et al. (2003) | 4 | 3.5 | 1.5 | 9 |
| Reinecke et al. (2014) | 4 | 4 | 2 | 10 |
| Ritchey et al.(2011) | 3 | 4 | 2 | 9 |
| Sakai et al.(2006) | 4 | 4 | 2 | 10 |
| Sankar et al. (2015) | 4 | 4 | 2 | 10 |
| Schienle et al. (2007) | 3.5 | 4 | 2 | 9.5 |
| Straube et al. (2006) | 4 | 4 | 2 | 10 |
| Tiger et al. (2014) | 4 | 4 | 1.5 | 9.5 |
| Yang et al. (2018) | 3.5 | 4 | 2 | 9.5 |


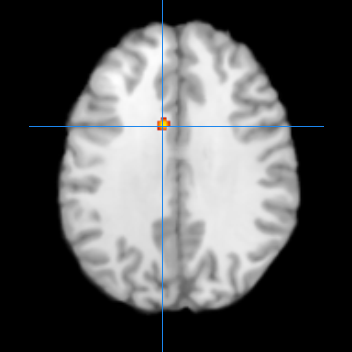

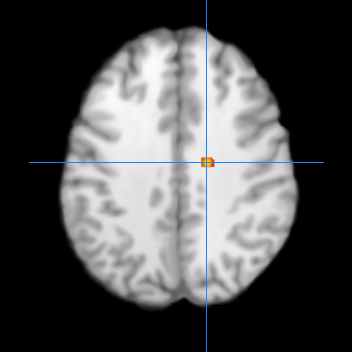

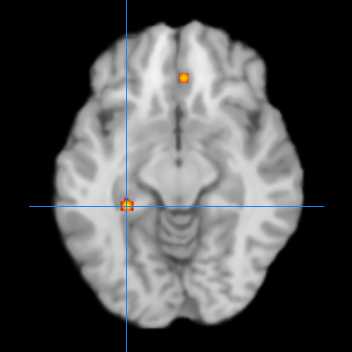

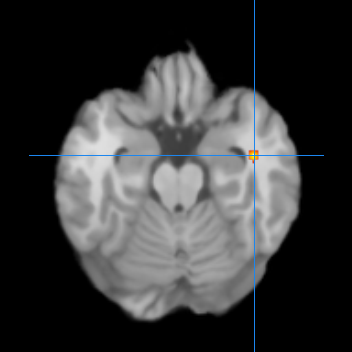

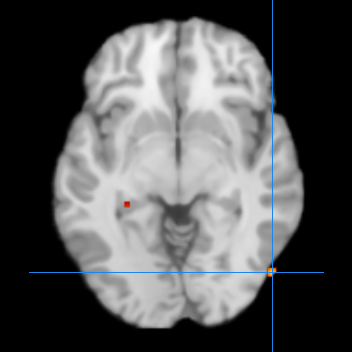

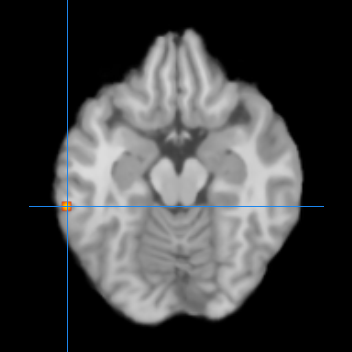

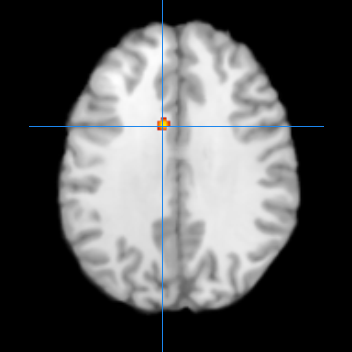


**Supplementary Figure 1**. Graph of data meta-analysis results for MDD in resting status. Uncorrected p＜0.001, min. cluster size = 200 mm³, the brain regions activated in the figure reached significant activation levels. The red activation area is the activity increase area. The brain regions activated in the diagram are, from left to right: row 1: the right Ventral anterior cingulate cortex (BA24), the left Ventral anterior cingulate cortex (BA24), the Parahippocampal Gyrus; row 2: the right Sub-Gyral (BA21), the left Inferior Temporal Gyrus (BA20), and the right Inferior Occipital Gyrus (BA19)

.


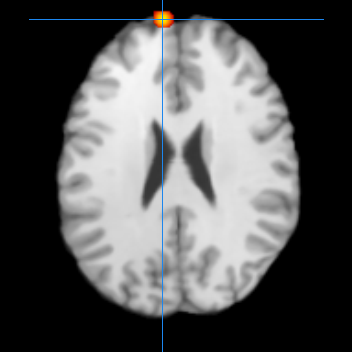

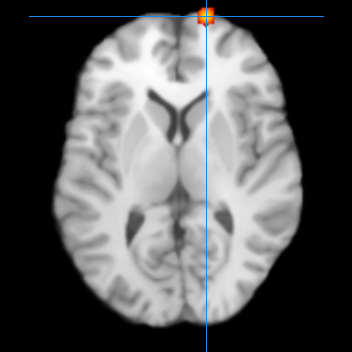

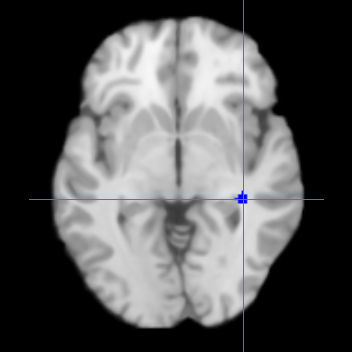


**Supplementary Figure 2.** Graph of data meta-analysis results for AD in resting status. Uncorrected p＜0.001, min. cluster size = 200 mm³, the brain regions activated in the figure reached significant activation levels. The red activation area is the activity increase area and the blue activation area is the activity decrease area. The brain regions activated in the diagram are, from left to right: the right Superior Frontal Gyrus (BA10), the left Superior Frontal Gyrus (BA9), the Caudate.


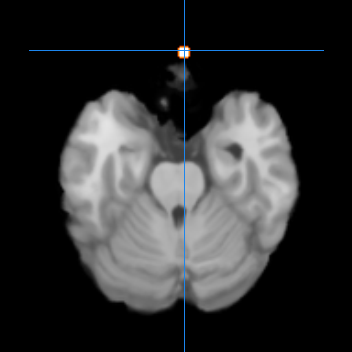

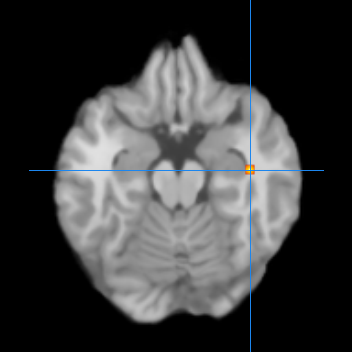

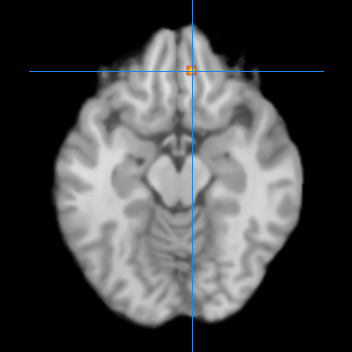

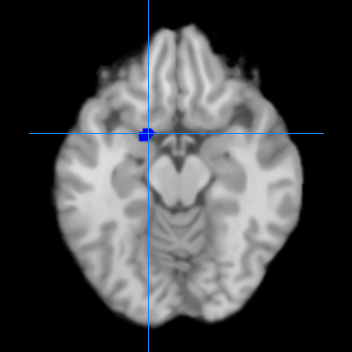

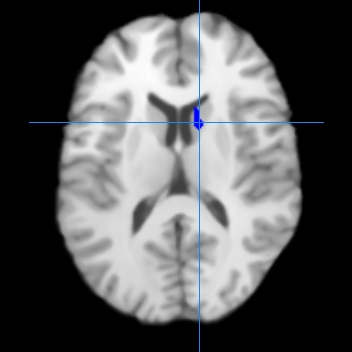

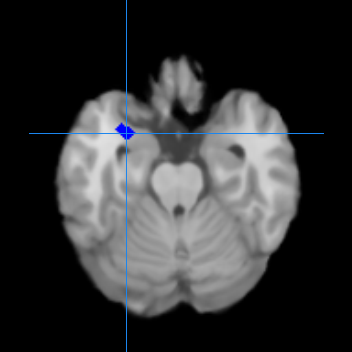


**Supplementary Figure 3.** Graph of data meta-analysis results for AD in task status. Uncorrected p＜0.001, min. cluster size = 200 mm³, the brain regions activated in the figure reached significant activation levels. The red activation area is the activity increase area and the blue activation area is the activity decrease area. The brain regions activated in the diagram are, from left to right: row 1: the right Orbital Frontal Lobe (BA11), the right Parahippocampal Gyrus, the right Dorsal anterior cingulate cortex (BA32); row 2: the left Lentiform Nucleus, the right Caudate, and the left Dorsal entorhinal cortex (BA34).
